# Data on cardiovascular and pulmonary diseases among smokers of menthol and non-menthol cigarettes compiled from the National Health and Nutrition Examination Survey (NHANES), 1999–2012

**DOI:** 10.1016/j.dib.2017.04.021

**Published:** 2017-04-20

**Authors:** Cynthia Van Landingham, William Fuller, Greg Mariano, Kristin Marano, Geoffrey Curtin, Sandra I. Sulsky

**Affiliations:** a Ramboll Environ, 3107 Armand Monroe, LA 71201, United States; b Ramboll Environ US Corporation, 4350 North Fairfax Drive, Suite 300, Arlington, VA 22203, United States; c RAI Services Company, 401 North Main Street, P.O. Box 464, Winston-Salem, NC 27102, United States; d Ramboll Environ US Corporation, 28 Amity Street, Suite 2A, Amherst, MA 01002, United States

**Keywords:** NHANES, Menthol versus non-menthol, Survey methods, Reanalysis, Cross-model validation

## Abstract

This Data in Brief contains results from three different survey logistic regression models comparing risks of self-reported diagnoses of cardiovascular and pulmonary diseases among smokers of menthol and non-menthol cigarettes. Analyses employ data from National Health and Nutrition Examination Survey (NHANES) cycles administered between 1999 and 2012, combined and in subsets. Raw data may be downloaded from the National Center for Health Statistics. Results were not much affected by which covariates were included in the models, but depended strongly on the NHANES cycles included in the analysis. All three models returned elevated risk estimates for three endpoints when they were run in individual NHANES cycles (congestive heart failure in 2001–02; hypertension in 2003–04; and chronic obstructive pulmonary disease in 2005–06), and all three models returned null results for these endpoints when data from 1999–2012 were combined.

**Specifications Table**

**Table:** 

Subject area	Epidemiology
More specific subject area	Health risks associated with smoking menthol vs. non-menthol cigarettes
Type of data	Tables
How data was acquired	Downloaded from US National Center for Health Statistics and analyzed using survey logistic regression methods
Data format	Analyzed
Experimental factors	None
Experimental features	Self-reported diagnoses of cardiovascular and pulmonary diseases are compared for smokers of menthol and non-menthol cigarettes
Data source location	USA
Data accessibility	Data are available from the US National Center for Health Statistics http://www.cdc.gov/nchs/nhanes/nhanes_questionnaires.htm

**Value of the data**

• Results of different models run on the same data set provide insights into how the data (i.e., which cycles of NHANES) and the covariates selected for inclusion in a model influence risk estimates.
• Estimates based on individual (i.e., 2-year) cycles of the NHANES versus estimates from combined cycles of NHANES show inconsistency and illustrate that analyses using individual cycles should not be used to draw causal inferences about the population.
• The data provided here allow comparisons between analyses published in two recent papers that reported contradictory results.

## 1 Experimental design, materials and methods

Two recent publications reported contradictory findings from analyses of data from the National Health and Nutrition Examination Survey (NHANES). Vozoris reported a statistically significantly increased adjusted odds of stroke diagnosis among menthol compared with non-menthol cigarette smokers, in particular among non-African Americans, using data from 2007–2008 cycle (incorrectly reported as 2001–2008) of NHANES [5]. Rostron did not detect a difference in stroke risk among smokers of menthol compared with non-menthol cigarettes, based on analyses of NHANES data from the 1999 through 2010 cycles [3]. Our investigation of the reasons for the discordant results reported by Vozoris and Rostron with respect to stroke risk, and the results of new analyses comparing stroke risks among smokers of menthol and non-menthol cigarettes that use all NHANES cycles from 1999 through 2012 is available elsewhere [4]. The differences between the Vozoris [5] and Rostron [3] results were shown to be mainly due to the inadvertent exclusion of all but the 2007–2008 NHANES data from the Vozoris [5] analysis. The data presented here examine risks of other endpoints evaluated by Vozoris (i.e., hypertension (HTN), myocardial infarction (MI), congestive heart failure (CHF), and chronic obstructive pulmonary disease(COPD)) among smokers of menthol compared with non-menthol cigarettes estimated according to three different logistic regression models: 1) models proposed by Vozoris, using NHANES 2007–2008, 1999–2010, and 1999–2012; 2) models proposed by Rostron, using NHANES 2007–2008, 1999–2010, and 1999–2012; and 3) a new set of models we developed with purposeful selection techniques using NHANES 1999–2012.

NHANES is a nationally representative survey of US, non-institutionalized civilians. It is conducted in two year cycles, with approximately 10,000 individuals in each cycle. Interviews elicit information on demographic characteristics (e.g., age, gender, race/ethnicity), smoking habits, and whether a health professional had ever diagnosed the participant with certain medical conditions. Cycles of the NHANES can be combined, or they can be analyzed individually. Because NHANES employs a complex, multistage, sampling strategy, survey statistics must be used to analyze the data and to generalize findings to the US population. In this case, we used the SURVEYLOGISTIC procedure of SAS/STAT© version 9.4 to perform logistic regression accounting for the complex sampling design, i.e., using both the masked variance pseudo-primary sampling unit (SMDVPSU) and the masked variance pseudo-stratum (SDMVSTRA) variables, using the adjusted 2 year interview weight (WTINT2YR), and using Taylor series linearization to estimate the covariance matrix. Weights were adjusted for the inclusion of multiple surveys [2] by dividing the WTINT2YR variable by the number of cycles used in each analysis. We additionally ran all models within strata defined by age, race/ethnicity, and gender using the SAS DOMAIN statement to specify these subpopulations and to ensure the variance and standard errors were calculated correctly. See associated file SAS CODE.DOCX for the code to combine the cycles of NHANES with common variables and an example of the Proc Logistic code used for analysis.

Following both Vozoris and Rostron, we defined current smokers as those who had smoked ≥1 of the last 30 days and who were ≥20 years old at the time of the interview. Table 1 shows the variables we used in these analyses. We identified cases by their self-reported diagnoses according to the question “has a doctor or other health professional ever told you that you had [high blood pressure, a heart attack, congestive heart failure, a stroke, or COPD (emphysema or chronic bronchitis)]” (yes/no). We considered all other responses to be a non-response and set them as missing. Stroke was the subject of Van Landingham et al. [4], and data are not presented here.

We ran three sets of models for each outcome using data from NHANES 2007 to 2008 (as used by Vozoris), from 1999 to 2010 (as used by Rostron) and from 1999 to 2012 (all cycles available when we undertook the project) to determine if the selection of covariates or cycles of the NHANES influenced the results. First, we implemented the model described by Vozoris (Table 2, Table 3, Table 4); second, we implemented the model described by Rostron (Table 5, Table 6, Table 7); last, we developed a new model for each outcome using purposeful selection of covariates (Table 8). Purposeful selection of covariates was conducted as follows: a preliminary model consisted of cigarette type (menthol or non-menthol) and all relevant, potential covariates (Table 1) with cigarette type forced to remain in all models. We identified each covariate, other than cigarette type, with a *p*-value of greater than 0.05. We refit the model after dropping the covariate with the largest *p*-value, until only cigarette type and covariates with *p*-values of 0.05 or less remained. Each covariate that had been dropped was added back individually, and we calculated the relative percent change in the regression coefficient for cigarette type for the larger model compared with the model containing only statistically significant covariates (Eq. (1)). If including a given covariate resulted in a relative percent change in the regression coefficient greater than 15%, that covariate was retained in the model.

(1) relative%change=|1−originalestimate/newestimate|×100

**Fig. 1:**
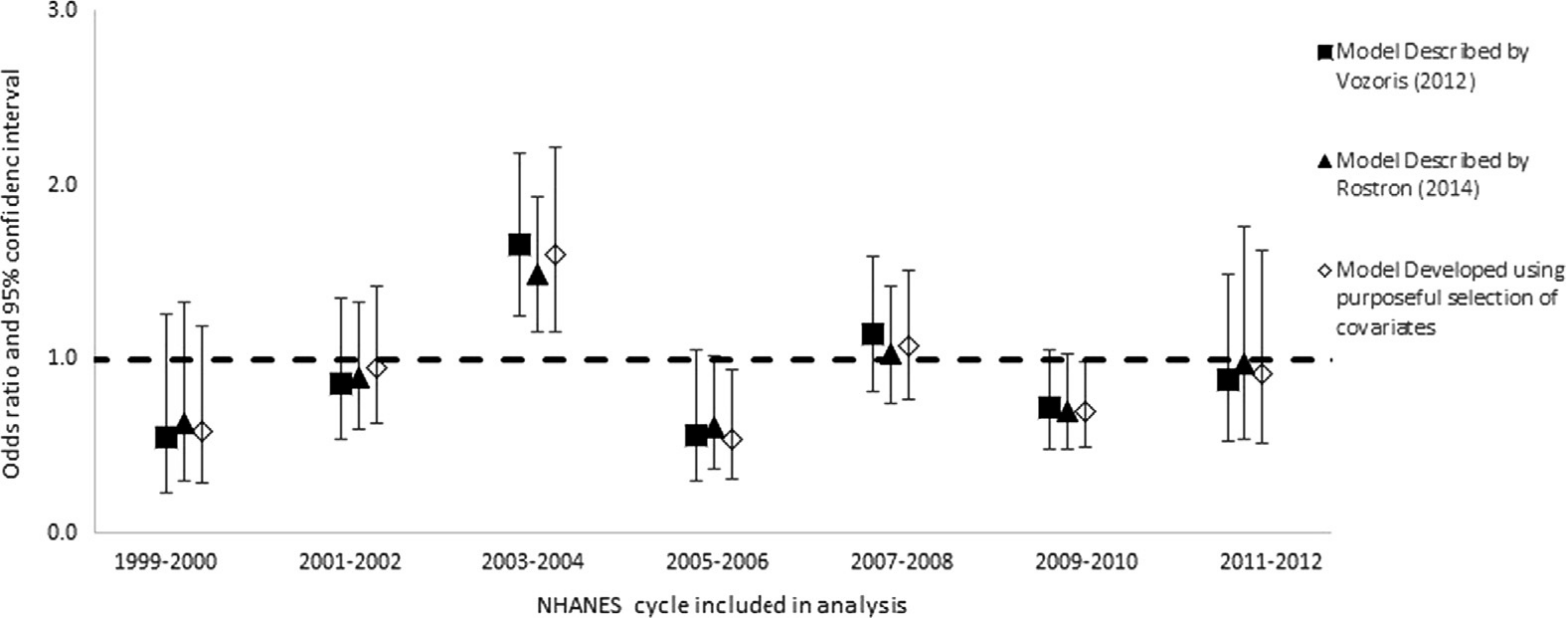
Odds ratios and 95% confidence intervals: risk of hypertension among all smokers of menthol vs. non-menthol cigarettes according to three different models, individual cycles of the NHANES from 1999 through 2012.

**Fig. 2:**
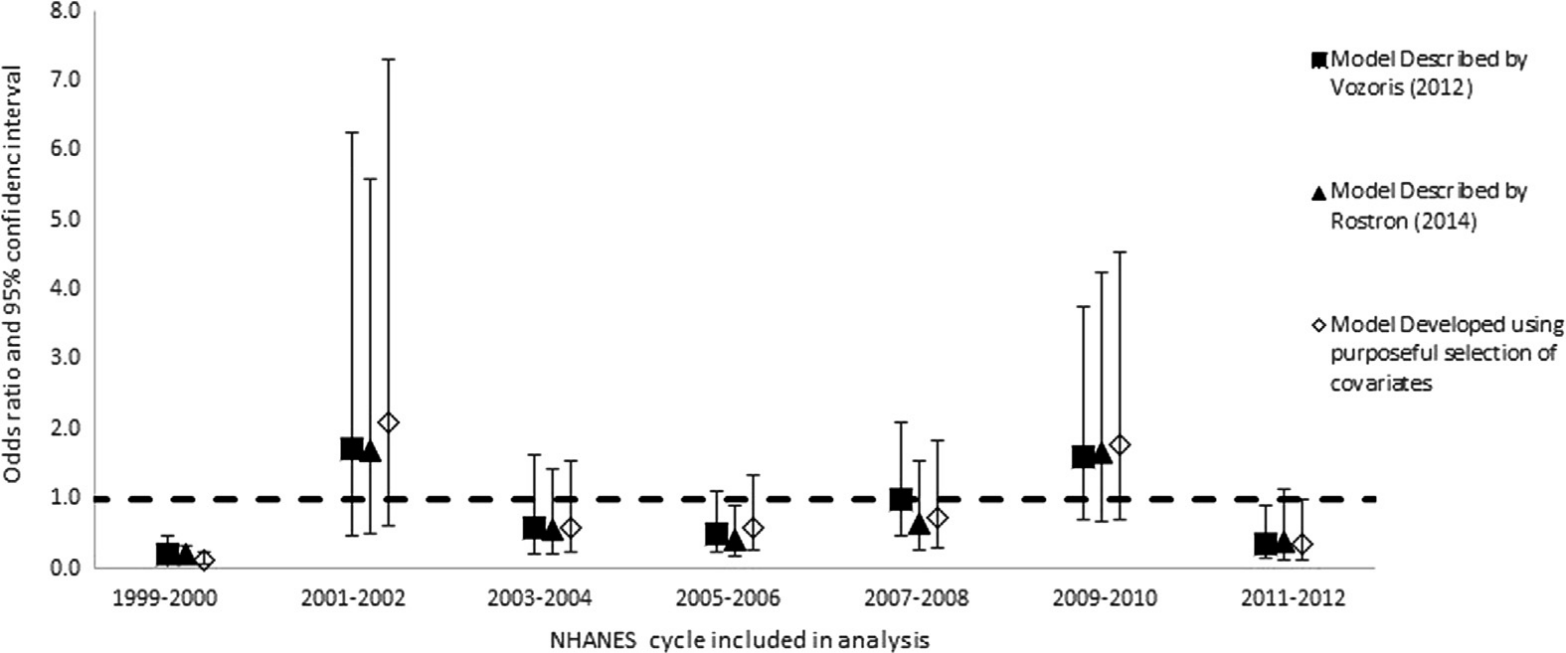
Odds ratios and 95% confidence intervals: risk of myocardial infarction among all smokers of menthol vs. non-menthol cigarettes according to three different models, individual cycles of the NHANES from 1999 through 2012.

**Fig. 3:**
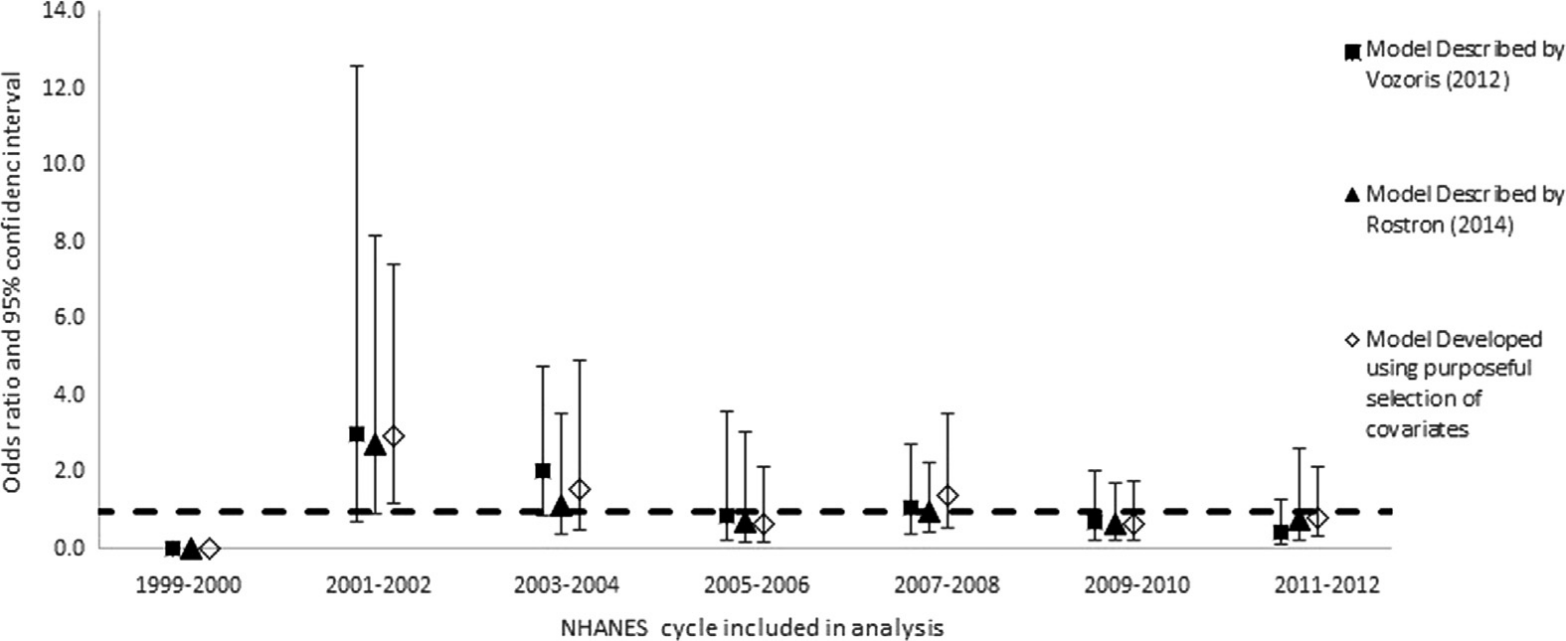
Odds ratios and 95% confidence intervals: risk of congestive heart failure among all smokers of menthol vs. non-menthol cigarettes according to three different models, individual cycles of the NHANES from 1999 through 2012.

**Fig. 4:**
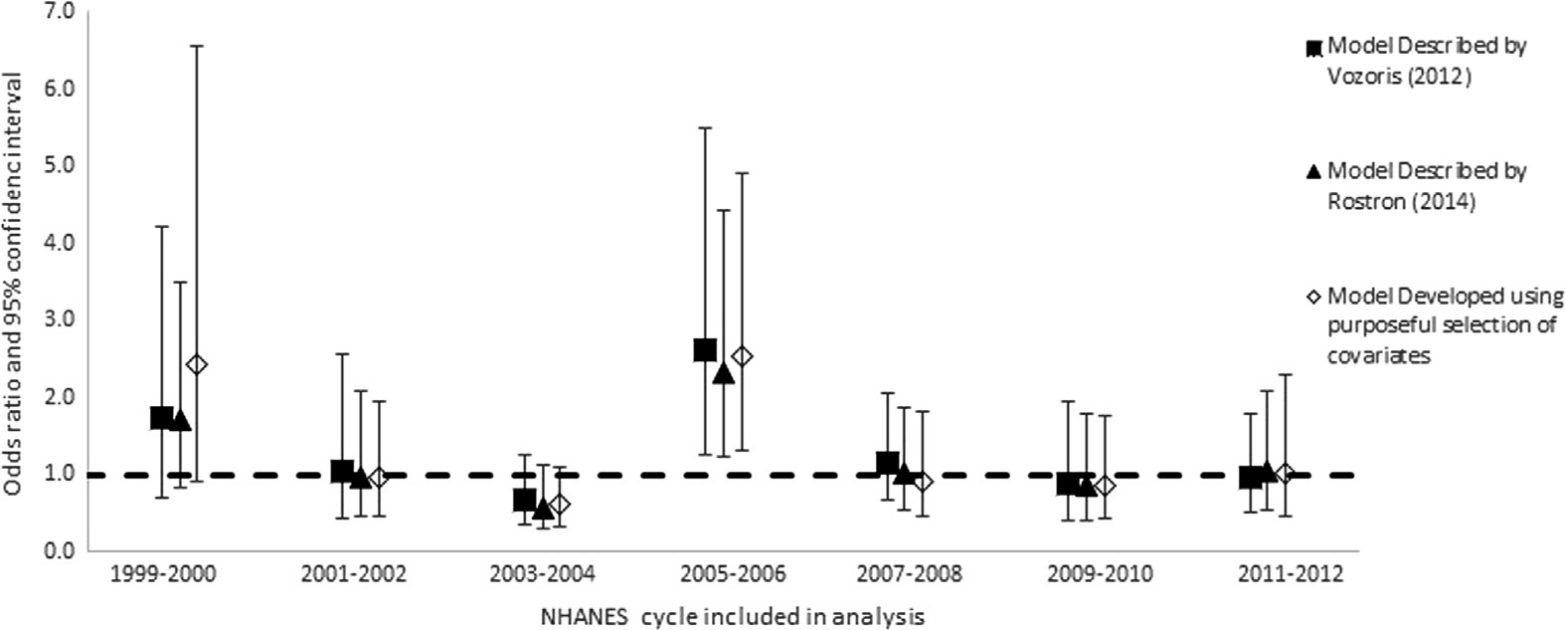
Odds ratios and 95% confidence intervals: Risk of chronic obstructive pulmonary disease among all smokers of menthol vs. non-menthol cigarettes according to three different models, individual cycles of the NHANES from 1999 through 2012.

Once we determined the covariates to include in the model (main effects), we explored all the possible interactions between the covariates (excluding cigarette type). We added all interaction terms with *p*-values less than or equal to 0.1 to the model individually, along with the main effect terms, and retained them if the relevant coefficients in the fully adjusted model were statistically significant, with *p*-values of 0.05 or less. We retained statistically significant interaction terms in the model only if one or both main effects were also statistically significant. We used domain variables to define strata according to race/ethnicities, genders, and age groups, but did not repeat the model building process. We then re-ran each model for individual cycles of the NHANES in order to determine if there were anomalous or secular patterns in risk of any outcome that might be overlooked in the combined analysis (Fig. 1, Fig. 2, Fig. 3, Fig. 4).

## Figures and Tables

**Table 1:** NHANES variables considered in analyses.

**NHANES variable**	**Description**
RIDAGEYR	Age
RIAGENDR	Gender
RIDRETH1	Race (races were combined as African American (i.e., non-Hispanic Black) or non-African American (i.e., Mexican-American, other Hispanic, non-Hispanic white and Other Races). When non-Hispanic Black, non-Hispanic White and Mexican Americans were reported, the “other Hispanic” and “other races” were combined into a category (Other). The Other category was not reported separately.
SMD070	Average # of cigarettes smoked per day
SMD080, SMD641	# days smoked in last 30 days. Data were captured in the variable SMD080 in NHANES 1999–2000 and 2001–2002, and in SMD641 starting in 2003.
SMD030	Age started smoking
BMXBMI	Body Mass Index
INDFMPIR	Poverty to Income Ratio (PIR)
DMDEDUC2^a^	Highest education level
INDHHINC, INDHHIN2^a^	Household Income. Data were captured in the variable INDHHINC in NHANES surveys before 2007, and in INDHHIN2 from 2007 through 2012.
BPQ020	Hypertension
MCQ160E	Myocardial Infarction
MCD160B	Congestive Heart Failure
MCQ160F	Stroke
MCQ160G, MCQ160K	Chronic Obstructive Pulmonary Disease (a yes for either variable indicated a yes for COPD)
SMD075	# of years smoked
SMQ140, SMQ170, SMQ200, SMD2130	Used other tobacco products (a yes for any of these four codes indicated a yes for Used other tobacco products)

Calculated variables	
Pack years^b^	Eq. 1. Average # of cigarettes smoked per day/20×(Age−Age started smoking+1)
	Eq. 2. Average # of cigarettes smoked per day/20 × # of years smoked
	Eq. 3. Average # of cigarettes smoked per day/20

a Values indicating “do not know” and “refused” for these variables were retained in the analyses.

b Pack years were calculated using Eq. (1), unless age started smoking was missing. Eq. (2) was used if age started smoking was missing and # of years smoked was available. Eq. (3) was used only as a last resort when only average # of cigarettes per day was available.

**Table 2:** Model specified by Vozoris [5]^a^ using data from NHANES 2007–2008; unweighted counts, adjusted odds ratios (AOR) and 95% confidence intervals (CI).

**Stratum**	**Diagnosis**^b^	**Cigarette preference**	**Cases**	**Non-Cases**	**AOR**	**95% CI**		**Total *N***
						**Lower**	**Upper**	
All	HTN	Non-Menthol	225	583				1158
		Menthol	126	224	1.14	0.82	1.59	
	MI	Non-Menthol	40	766				1156
		Menthol	12	338	0.99	0.47	2.10	
	CHF	Non-Menthol	20	785				1155
		Menthol	11	339	1.06	0.41	2.75	
	COPD	Non-Menthol	105	704				1159
		Menthol	37	313	1.17	0.66	2.05	
Female	HTN	Non-Menthol	97	219				506
		Menthol	77	113	1.30	0.75	2.25	
	MI	Non-Menthol	14	301				505
		Menthol	6	184	0.53	0.17	1.63	
	CHF	Non-Menthol	7	308				505
		Menthol	5	185	0.63	0.15	2.67	
	COPD	Non-Menthol	59	257				506
		Menthol	29	161	1.04	0.62	1.75	
Male	HTN	Non-Menthol	128	364				652
		Menthol	49	111	1.06	0.67	1.67	
	MI	Non-Menthol	26	465				651
		Menthol	6	154	1.55	0.41	5.85	
	CHF	Non-Menthol	13	477				650
		Menthol	6	154	0.96	0.28	3.29	
	COPD	Non-Menthol	46	447				653
		Menthol	8	152	1.68	0.45	6.31	
African American	HTN	Non-Menthol	36	67				278
		Menthol	71	104	1.84	0.72	4.72	
	MI	Non-Menthol	4	100				279
		Menthol	8	167	1.44	0.43	4.91	
	CHF	Non-Menthol	3	101				279
		Menthol	9	166	1.84	0.17	20.38	
	COPD	Non-Menthol	11	93				279
		Menthol	12	163	0.34	0.06	1.86	
Non-African American	HTN	Non-Menthol	189	516				880
		Menthol	55	120	1.02	0.66	1.57	
	MI	Non-Menthol	36	666				877
		Menthol	4	171	0.74	0.22	2.57	
	CHF	Non-Menthol	17	684				876
		Menthol	2	173	0.69	0.16	3.04	
	COPD	Non-Menthol	94	611				880
		Menthol	25	150	1.30	0.72	2.34	
Ages ≥70 years	HTN	Non-Menthol	32	24				69
		Menthol	11	2	0.11	0.00	11.20	
	MI	Non-Menthol	11	45				69
		Menthol	2	11	<0.001	<0.001	<0.001	
	CHF	Non-Menthol	3	50				66
		Menthol	1	12	<0.001	<0.001	<0.001	
	COPD	Non-Menthol	16	40				69
		Menthol	3	10	1.88	0.14	26.05	
Ages 20 to <70 years	HTN	Non-Menthol	193	559				1089
		Menthol	115	222	1.02	0.77	1.37	
	MI	Non-Menthol	29	721				1087
		Menthol	10	327	0.65	0.27	1.56	
	CHF	Non-Menthol	17	735				1089
		Menthol	10	327	0.68	0.25	1.83	
	COPD	Non-Menthol	89	664				1090
		Menthol	34	303	1.06	0.58	1.94	

a Model controls for age, gender, race/ethnicity, body mass index, total household income, average number of cigarettes smoked per day in the last 30 days, number of days smoked in the last 30 days, and age started smoking. Vozoris [5].

b HTN: hypertension; MI: myocardial infarction; CHF: congestive heart failure; COPD: chronic obstructive pulmonary disease.

**Table 3:** Model specified by Vozoris [5]^a^ using data from NHANES 1999–2010; unweighted counts, adjusted odds ratios (AOR) and 95% confidence intervals (CI).

**Stratum**	**Diagnosis**^b^	**Cigarette preference**	**Cases**	**Non-Cases**	**AOR**	**95% CI**		**Total *N***
						**Lower**	**Upper**	
All	HTN	Non-Menthol	1053	2934				5771
		Menthol	520	1264	0.90	0.75	1.08	
	MI	Non-Menthol	196	3810				5796
		Menthol	63	1727	0.97	0.64	1.47	
	CHF	Non-Menthol	111	3888				5788
		Menthol	46	1743	1.08	0.66	1.75	
	COPD	Non-Menthol	453	3562				5806
		Menthol	181	1610	1.25	0.92	1.69	
Female	HTN	Non-Menthol	461	1178				2552
		Menthol	285	628	0.88	0.67	1.16	
	MI	Non-Menthol	72	1569				2556
		Menthol	21	894	0.69	0.35	1.38	
	CHF	Non-Menthol	41	1598				2553
		Menthol	16	898	0.93	0.42	2.07	
	COPD	Non-Menthol	265	1379				2559
		Menthol	115	800	1.11	0.81	1.52	
Male	HTN	Non-Menthol	592	1756				3219
		Menthol	235	636	0.92	0.69	1.22	
	MI	Non-Menthol	124	2241				3240
		Menthol	42	833	1.28	0.77	2.13	
	CHF	Non-Menthol	70	2290				3235
		Menthol	30	845	1.22	0.68	2.19	
	COPD	Non-Menthol	188	2183				3247
		Menthol	66	810	1.57	0.93	2.65	
African American	HTN	Non-Menthol	183	227				1355
		Menthol	314	631	0.94	0.70	1.26	
	MI	Non-Menthol	28	383				1360
		Menthol	29	920	0.65	0.32	1.32	
	CHF	Non-Menthol	21	389				1359
		Menthol	26	923	0.63	0.31	1.28	
	COPD	Non-Menthol	41	371				1361
		Menthol	71	878	0.65	0.39	1.08	
Non-African American	HTN	Non-Menthol	870	2707				4416
		Menthol	206	633	1.49	0.71	3.12	
	MI	Non-Menthol	168	3427				4436
		Menthol	34	807	1.05	0.67	1.65	
Non-African American	CHF	Non-Menthol	90	3499				4429
		Menthol	20	820	1.38	0.73	2.59	
	COPD	Non-Menthol	412	3191				4445
		Menthol	110	732	1.35	0.99	1.84	
Ages ≥70 years	HTN	Non-Menthol	157	135				367
		Menthol	43	32	0.63	0.30	1.32	
	MI	Non-Menthol	43	248				368
		Menthol	12	65	1.02	0.36	2.85	
	CHF	Non-Menthol	26	260				362
		Menthol	9	67	1.06	0.38	2.98	
	COPD	Non-Menthol	65	228				370
		Menthol	14	63	0.94	0.42	2.09	
Ages 20 to <70 years	HTN	Non-Menthol	896	2799				5404
		Menthol	477	1232	0.82	0.69	0.98	
	MI	Non-Menthol	153	3562				5428
		Menthol	51	1662	0.71	0.45	1.15	
	CHF	Non-Menthol	85	3628				5426
		Menthol	37	1676	0.82	0.45	1.49	
	COPD	Non-Menthol	388	3334				5436
		Menthol	167	1547	1.17	0.86	1.60	

a Model controls for age, gender, race/ethnicity, body mass index, total household income, average number of cigarettes smoked per day in the last 30 days, number of days smoked in the last 30 days, and age started smoking. Vozoris [5].

b HTN: hypertension; MI: myocardial infarction; CHF: congestive heart failure; COPD: chronic obstructive pulmonary disease.

**Table 4:** Model specified by Vozoris [5]^a^ using data from NHANES 1999–2012; unweighted counts, adjusted odds ratios (AOR) and 95% confidence intervals (CI).

**Stratum**	**Diagnosis**^b^	**Cigarette preference**	**Cases**	**Non-Cases**	**AOR**	**95% CI**		**Total *N***
						**Lower**	**Upper**	
All	HTN	Non-Menthol	1236	3345				6710
		Menthol	651	1478	0.91	0.77	1.08	
	MI	Non-Menthol	228	4373				6736
		Menthol	72	2063	0.84	0.56	1.24	
	CHF	Non-Menthol	128	4467				6727
		Menthol	54	2078	0.95	0.61	1.49	
	COPD	Non-Menthol	527	4084				6747
		Menthol	218	1918	1.20	0.91	1.56	
Female	HTN	Non-Menthol	522	1331				2918
		Menthol	348	717	0.91	0.70	1.19	
	MI	Non-Menthol	79	1776				2922
		Menthol	23	1044	0.63	0.32	1.25	
	CHF	Non-Menthol	48	1805				2919
		Menthol	18	1048	0.76	0.36	1.61	
	COPD	Non-Menthol	297	1561				2925
		Menthol	140	927	1.11	0.83	1.48	
Male	HTN	Non-Menthol	714	2014				3792
		Menthol	303	761	0.91	0.69	1.21	
	MI	Non-Menthol	149	2597				3814
		Menthol	49	1019	1.07	0.67	1.71	
	CHF	Non-Menthol	80	2662				3808
		Menthol	36	1030	1.17	0.70	1.96	
	COPD	Non-Menthol	230	2523				3822
		Menthol	78	991	1.35	0.85	2.13	
African American	HTN	Non-Menthol	224	276				1639
		Menthol	407	732	0.97	0.73	1.28	
	MI	Non-Menthol	35	466				1644
		Menthol	34	1109	0.60	0.33	1.11	
	CHF	Non-Menthol	24	476				1642
		Menthol	31	1111	0.56	0.29	1.08	
	COPD	Non-Menthol	47	455				1645
		Menthol	90	1053	0.73	0.46	1.16	
Non-African American	HTN	Non-Menthol	1012	3069				5071
		Menthol	244	746	0.91	0.75	1.12	
	MI	Non-Menthol	193	3907				5092
		Menthol	38	954	0.88	0.56	1.38	
Non-African American	CHF	Non-Menthol	104	3991				5085
		Menthol	23	967	1.14	0.64	2.02	
	COPD	Non-Menthol	480	3629				5102
		Menthol	128	865	1.26	0.95	1.67	
Ages ≥70 years	HTN	Non-Menthol	180	153				421
		Menthol	55	33	0.81	0.41	1.62	
	MI	Non-Menthol	49	283				422
		Menthol	14	76	1.08	0.43	2.71	
	CHF	Non-Menthol	30	297				416
		Menthol	10	79	1.02	0.38	2.74	
	COPD	Non-Menthol	73	261				424
		Menthol	16	74	0.88	0.42	1.83	
Ages 20 to <70 years	HTN	Non-Menthol	1056	3192				6289
		Menthol	596	1445	0.82	0.69	0.96	
	MI	Non-Menthol	179	4090				6314
		Menthol	58	1987	0.60	0.39	0.93	
	CHF	Non-Menthol	98	4170				6311
		Menthol	44	1999	0.71	0.41	1.23	
	COPD	Non-Menthol	454	3823				6323
		Menthol	202	1844	1.11	0.84	1.46	

a Model controls for age, gender, race/ethnicity, body mass index, total household income, average number of cigarettes smoked per day in the last 30 days, number of days smoked in the last 30 days, and age started smoking. Vozoris [5].

b HTN: hypertension; MI: myocardial infarction; CHF: congestive heart failure; COPD: chronic obstructive pulmonary disease.

**Table 5:** Model specified by Rostron [3]^a^ using data from NHANES 2007–2008; unweighted counts, adjusted odds ratios (AOR), and 95% confidence intervals (CI).

**Stratum**	**Diagnosis**^b^	**Cigarette preference**	**Cases**	**Non-Cases**	**AOR**	**95% CI**		**Total *N***
						**Lower**	**Upper**	
All	HTN	Non-Menthol	215	546				1085
		Menthol	113	211	1.03	0.74	1.42	
	MI	Non-Menthol	39	720				1083
		Menthol	10	314	0.65	0.28	1.54	
	CHF	Non-Menthol	18	740				1082
		Menthol	9	315	0.98	0.43	2.26	
	COPD	Non-Menthol	99	663				1086
		Menthol	32	292	1.02	0.55	1.88	
Female	HTN	Non-Menthol	96	208				480
		Menthol	72	104	1.20	0.68	2.12	
	MI	Non-Menthol	14	289				479
		Menthol	5	171	0.34	0.10	1.15	
	CHF	Non-Menthol	5	298				479
		Menthol	5	171	0.91	0.27	3.10	
	COPD	Non-Menthol	55	249				480
		Menthol	26	150	1.01	0.56	1.82	
Male	HTN	Non-Menthol	119	338				605
		Menthol	41	107	0.83	0.50	1.37	
	MI	Non-Menthol	25	431				604
		Menthol	5	143	1.19	0.22	6.34	
	CHF	Non-Menthol	13	442				603
		Menthol	4	144	0.79	0.22	2.90	
	COPD	Non-Menthol	44	414				606
		Menthol	6	142	1.07	0.30	3.79	
Non-Hispanic Black	HTN	Non-Menthol	35	56				257
		Menthol	65	101	1.54	0.49	4.85	
	MI	Non-Menthol	4	88				258
		Menthol	8	158	1.25	0.51	3.07	
	CHF	Non-Menthol	3	89				258
		Menthol	8	158	2.35	0.39	14.09	
	COPD	Non-Menthol	10	82				258
		Menthol	11	155	0.49	0.09	2.66	
Non-Hispanic White	HTN	Non-Menthol	134	327				559
		Menthol	29	69	0.91	0.56	1.48	
	MI	Non-Menthol	29	430				557
		Menthol	1	97	0.32	0.02	4.26	
Non-Hispanic White	CHF	Non-Menthol	11	447				556
		Menthol	0	98	<0.001	<0.001	<0.001	
	COPD	Non-Menthol	71	390				559
		Menthol	12	86	1.04	0.50	2.18	
Mexican American	HTN	Non-Menthol	28	86				132
		Menthol	7	11	1.67	0.46	6.03	
	MI	Non-Menthol	2	111				131
		Menthol	0	18	<0.001	<0.001	<0.001	
	CHF	Non-Menthol	3	110				131
		Menthol	0	18	<0.001	<0.001	<0.001	
	COPD	Non-Menthol	5	109				132
		Menthol	2	16	2.50	0.41	15.42	
Ages ≥70 years	HTN	Non-Menthol	32	23				65
		Menthol	8	2	1.45	0.22	9.70	
	MI	Non-Menthol	12	43				65
		Menthol	0	10	<0.001	<0.001	<0.001	
	CHF	Non-Menthol	3	49				62
		Menthol	0	10	<0.001	<0.001	<0.001	
	COPD	Non-Menthol	16	39				65
		Menthol	1	9	1.05	0.09	12.30	
Ages 20 to <70 years	HTN	Non-Menthol	183	523				1020
		Menthol	105	209	1.04	0.76	1.43	
	MI	Non-Menthol	27	677				1018
		Menthol	10	304	0.76	0.35	1.65	
	CHF	Non-Menthol	15	691				1020
		Menthol	9	305	0.97	0.39	2.46	
	COPD	Non-Menthol	83	624				1021
		Menthol	31	283	1.08	0.57	2.04	

a Model controls for: age, gender, race/ethnicity, body mass index, PIR, and pack-years of smoking. Rostron [3].

b HTN: hypertension; MI: myocardial infarction; CHF: congestive heart failure; COPD: chronic obstructive pulmonary disease

**Table 6:** Model specified by Rostron [3]^a^ using data from NHANES 1999–2010; unweighted counts, adjusted odds ratios (AOR) and 95% confidence intervals (CI).

**Stratum**	**Diagnosis**^b^	**Cigarette preference**	**Cases**	**Non-Cases**	**AOR**	**95% CI**		**Total *N***
						**Lower**	**Upper**	
All	HTN	Non-Menthol	1029	2935				5731
		Menthol	510	1257	0.87	0.73	1.03	
	MI	Non-Menthol	191	3799				5763
		Menthol	59	1714	0.82	0.53	1.25	
	CHF	Non-Menthol	105	3876				5753
		Menthol	43	1729	1.00	0.62	1.63	
	COPD	Non-Menthol	441	3557				5772
		Menthol	169	1605	1.14	0.85	1.52	
Female	HTN	Non-Menthol	457	1173				2539
		Menthol	285	624	0.87	0.65	1.15	
	MI	Non-Menthol	71	1562				2544
		Menthol	20	891	0.62	0.30	1.31	
	CHF	Non-Menthol	37	1593				2540
		Menthol	16	894	0.96	0.42	2.23	
	COPD	Non-Menthol	262	1373				2546
		Menthol	108	803	1.03	0.75	1.42	
Male	HTN	Non-Menthol	572	1762				3192
		Menthol	225	633	0.87	0.65	1.17	
	MI	Non-Menthol	120	2237				3219
		Menthol	39	823	1.04	0.60	1.80	
	CHF	Non-Menthol	68	2283				3213
		Menthol	27	835	1.07	0.60	1.91	
	COPD	Non-Menthol	179	2184				3226
		Menthol	61	802	1.42	0.84	2.39	
Non-Hispanic Black	HTN	Non-Menthol	176	215				1332
		Menthol	311	630	0.93	0.68	1.27	
	MI	Non-Menthol	28	364				1337
		Menthol	27	918	0.53	0.27	1.03	
	CHF	Non-Menthol	20	371				1336
		Menthol	24	921	0.61	0.30	1.26	
	COPD	Non-Menthol	37	356				1338
		Menthol	68	877	0.70	0.41	1.20	
Non-Hispanic White	HTN	Non-Menthol	609	1806				2980
		Menthol	126	439	0.85	0.69	1.06	
Non-Hispanic White	MI	Non-Menthol	129	2288				2983
		Menthol	22	544	0.85	0.50	1.42	
	CHF	Non-Menthol	63	2352				2979
		Menthol	14	550	1.20	0.60	2.40	
	COPD	Non-Menthol	321	2101				2988
		Menthol	75	491	1.18	0.84	1.66	
Mexican American	HTN	Non-Menthol	154	607				877
		Menthol	36	80	1.34	0.68	2.63	
	MI	Non-Menthol	18	764				899
		Menthol	4	113	2.82	0.36	22.11	
	CHF	Non-Menthol	16	762				895
		Menthol	2	115	0.46	0.11	1.95	
	COPD	Non-Menthol	42	741				900
		Menthol	6	111	0.69	0.27	1.76	
Ages ≥70 years	HTN	Non-Menthol	150	129				351
		Menthol	41	31	0.70	0.39	1.27	
	MI	Non-Menthol	44	234				352
		Menthol	10	64	0.78	0.29	2.13	
	CHF	Non-Menthol	25	247				345
		Menthol	8	65	0.86	0.30	2.46	
	COPD	Non-Menthol	64	216				354
		Menthol	11	63	0.76	0.35	1.66	
Ages 20 to <70 years	HTN	Non-Menthol	879	2806				5380
		Menthol	469	1226	0.84	0.70	1.00	
	MI	Non-Menthol	147	3565				5411
		Menthol	49	1650	0.76	0.47	1.22	
	CHF	Non-Menthol	80	3629				5408
		Menthol	35	1664	0.89	0.49	1.65	
	COPD	Non-Menthol	377	3341				5418
		Menthol	158	1542	1.15	0.85	1.56	

a Model controls for: age, gender, race/ethnicity, body mass index, PIR, and pack-years of smoking. Rostron [3].

b HTN: Hypertension; MI: myocardial infarction; CHF: congestive heart failure; COPD: chronic obstructive pulmonary disease.

**Table 7:** Model specified by Rostron [3]^a^ using data from NHANES 1999–2012; unweighted counts, adjusted odds ratios (AOR) and 95% confidence intervals (CI).

**Stratum**	**Diagnosis**^b^	**Cigarette preference**	**Cases**	**Non-Cases**	**AOR**	**95% CI**		**Total *N***
						**Lower**	**Upper**	
All	HTN	Non-Menthol	1202	3319				6615
		Menthol	632	1462	0.89	0.75	1.06	
	MI	Non-Menthol	218	4330				6648
		Menthol	66	2034	0.73	0.49	1.10	
	CHF	Non-Menthol	121	4419				6637
		Menthol	51	2046	0.95	0.62	1.47	
	COPD	Non-Menthol	510	4047				6658
		Menthol	204	1897	1.12	0.86	1.45	
Female	HTN	Non-Menthol	515	1317				2888
		Menthol	345	711	0.89	0.68	1.18	
	MI	Non-Menthol	77	1758				2893
		Menthol	22	1036	0.60	0.29	1.22	
	CHF	Non-Menthol	44	1788				2889
		Menthol	19	1038	0.92	0.44	1.89	
	COPD	Non-Menthol	290	1547				2895
		Menthol	133	925	1.05	0.79	1.40	
Male	HTN	Non-Menthol	687	2002				3727
		Menthol	287	751	0.89	0.66	1.19	
	MI	Non-Menthol	141	2572				3755
		Menthol	44	998	0.88	0.53	1.47	
	CHF	Non-Menthol	77	2631				3748
		Menthol	32	1008	1.01	0.59	1.73	
	COPD	Non-Menthol	220	2500				3763
		Menthol	71	972	1.25	0.79	1.99	
Non-Hispanic Black	HTN	Non-Menthol	213	263				1597
		Menthol	395	726	0.97	0.72	1.29	
	MI	Non-Menthol	35	442				1602
		Menthol	30	1095	0.49	0.27	0.89	
	CHF	Non-Menthol	23	453				1600
		Menthol	28	1096	0.57	0.30	1.10	
	COPD	Non-Menthol	43	435				1603
		Menthol	85	1040	0.79	0.49	1.27	
Non-Hispanic White	HTN	Non-Menthol	705	2024				3377
		Menthol	149	499	0.86	0.69	1.07	
	MI	Non-Menthol	146	2584				3379
		Menthol	24	625	0.75	0.45	1.25	
Non-Hispanic White	CHF	Non-Menthol	74	2655				3376
		Menthol	18	629	1.13	0.64	1.99	
	COPD	Non-Menthol	373	2363				3385
		Menthol	90	559	1.17	0.86	1.60	
Mexican American	HTN	Non-Menthol	169	645				945
		Menthol	39	92	1.33	0.74	2.37	
	MI	Non-Menthol	19	816				967
		Menthol	4	128	2.41	0.33	17.75	
	CHF	Non-Menthol	17	814				963
		Menthol	2	130	0.39	0.07	2.11	
	COPD	Non-Menthol	44	792				968
		Menthol	7	125	0.61	0.27	1.36	
Ages ≥70 years	HTN	Non-Menthol	172	145				401
		Menthol	52	32	0.85	0.48	1.49	
	MI	Non-Menthol	49	267				402
		Menthol	12	74	1.01	0.42	2.44	
	CHF	Non-Menthol	28	282				395
		Menthol	9	76	0.96	0.33	2.73	
	COPD	Non-Menthol	72	246				404
		Menthol	13	73	0.87	0.42	1.79	
Ages 20 to <70 years	HTN	Non-Menthol	1030	3174				6214
		Menthol	580	1430	0.85	0.71	1.01	
	MI	Non-Menthol	169	4063				6246
		Menthol	54	1960	0.64	0.41	1.00	
	CHF	Non-Menthol	93	4137				6242
		Menthol	42	1970	0.81	0.48	1.37	
	COPD	Non-Menthol	438	3801				6254
		Menthol	191	1824	1.11	0.84	1.45	

a Model controls for: age, gender, race/ethnicity, body mass index, PIR, and pack-years of smoking. Rostron [3].

b HTN: hypertension; MI: myocardial infarction; CHF: congestive heart failure; COPD: chronic obstructive pulmonary disease.

**Table 8:** Analysis of NHANES 1999‐2012^a^; proportionate distribution of menthol and non-menthol cigarette preference, unweighted counts, adjusted odds ratios (AOR) and 95% confidence intervals (CI).

**Stratum**	**Diagnosis**^b^	**Cigarette preference**	**Cases**	**Non-Cases**	**AOR**	**95% CI**		**Total *N***
						**Lower**	**Upper**	
All	HTN^c^	Non-Menthol	1316	3623				7238
		Menthol	703	1596	0.91	0.76	1.07	
	MI^d^	Non-Menthol	218	4229				6509
		Menthol	66	1996	0.76	0.50	1.14	
	CHF^e^	Non-Menthol	122	4443				6671
		Menthol	51	2055	1.00	0.66	1.54	
	COPD^f^	Non-Menthol	507	3924				6486
		Menthol	202	1853	1.15	0.88	1.50	
Female	HTN^c^	Non-Menthol	557	1416				3137
		Menthol	381	783	0.89	0.68	1.16	
	MI^d^	Non-Menthol	77	1731				2844
		Menthol	22	1014	0.98	0.60	1.62	
	CHF^e^	Non-Menthol	44	1796				2902
		Menthol	19	1043	1.15	0.64	2.07	
	COPD^f^	Non-Menthol	288	1513				2832
		Menthol	133	898	1.09	0.81	1.47	
Male	HTN^c^	Non-Menthol	759	2207				4101
		Menthol	322	813	0.921	0.70	1.21	
	MI^d^	Non-Menthol	141	2498				3665
		Menthol	44	982	0.61	0.29	1.28	
	CHF^e^	Non-Menthol	78	2647				3769
		Menthol	32	1012	1.00	0.62	1.63	
	COPD^f^	Non-Menthol	219	2411				3654
		Menthol	69	955	1.24	0.78	1.95	
Non-Hispanic Black	HTN^c^	Non-Menthol	745	2146				3594
		Menthol	165	538	0.88	0.70	1.09	
	MI^d^	Non-Menthol	146	2549				3336
		Menthol	24	617	0.79	0.47	1.33	
	CHF^e^	Non-Menthol	75	2659				3383
		Menthol	18	631	1.14	0.62	2.13	
	COPD^f^	Non-Menthol	373	2322				3334
		Menthol	90	549	1.23	0.90	1.69	
Non-Hispanic White	HTN^c^	Non-Menthol	241	296				1768
		Menthol	441	790	0.97	0.74	1.29	
	MI^d^	Non-Menthol	35	434				1581
		Menthol	30	1082	0.50	0.28	0.91	
Non-Hispanic White	CHF^e^	Non-Menthol	23	453				1606
		Menthol	28	1102	0.51	0.26	0.99	
	COPD^f^	Non-Menthol	42	426				1574
		Menthol	83	1023	0.70	0.43	1.14	
Mexican American	HTN^c^	Non-Menthol	194	747				1091
		Menthol	44	106	1.01	0.61	1.68	
	MI^d^	Non-Menthol	20	772				911
		Menthol	4	115	2.11	0.35	12.87	
	CHF^e^	Non-Menthol	17	825				975
		Menthol	2	131	0.51	0.11	2.27	
	COPD^f^	Non-Menthol	44	739				902
		Menthol	7	112	0.62	0.27	1.41	
Ages ≥70 years	HTN^c^	Non-Menthol	191	166				454
		Menthol	61	36	0.98	0.52	1.83	
	MI^d^	Non-Menthol	49	268				403
		Menthol	12	74	1.11	0.49	2.48	
	CHF^e^	Non-Menthol	29	284				399
		Menthol	9	77	0.76	0.27	2.14	
	COPD^f^	Non-Menthol	71	243				399
		Menthol	13	72	0.80	0.35	1.80	
Ages 20 to <70 years	HTN^c^	Non-Menthol	1125	3457				6784
		Menthol	642	1560	0.91	0.76	1.08	
	MI^d^	Non-Menthol	169	3961				6106
		Menthol	54	1922	0.70	0.45	1.10	
	CHF^e^	Non-Menthol	93	4159				6272
		Menthol	42	1978	1.02	0.65	1.59	
	COPD^f^	Non-Menthol	436	3681				6087
		Menthol	189	1781	1.18	0.90	1.56	

a Models developed using purposeful selection of covariates [1]. The same covariates were included in the models run in the subdomains as were included in the model for the population, overall.

b MI: Myocardial infarction; CHF: congestive heart failure; COPD: chronic obstructive pulmonary disease.

c Odds of hypertension diagnosis controlling for age, gender, BMI, education, ethnicity, gender*ethnicity, BMI*education.

d Odds of myocardial infarction (MI) diagnosis controlling for age, age started smoking, PIR, education, race/ethnicity, BMI*PIR, race/ethnicity*education.

e Odds of congestive heart failure (CHF) diagnosis controlling for age, BMI, PIR, education, BMI*education.

f Odds of chronic obstructive pulmonary disease diagnosis controlling for age, gender, cigarettes smoked per day, days smoked in last 30, age started smoking, BMI, PIR, education, gender*days smoked in last 30, gender*race/ethnicity, education* days smoked in last 30, PIR*education, PIR*race/ethnicity.
