# Supplementary material for: Data on cardiovascular and pulmonary diseases among smokers of menthol and non-menthol cigarettes compiled from the National Health and Nutrition Examination Survey (NHANES), 1999–2012
Source: Data Brief. 2017 Apr 20;12:386–99. doi: 10.1016/j.dib.2017.04.021 (PMC5415547; doi:10.1016/j.dib.2017.04.021)
Supplement: Supplementary file 1 — Supplementary material [file mmc1.docx]

libname fin '.';

options nocenter;

proc format library=fin;

value yn 0 = 'No'

1 = 'Yes'

2 = 'No';

value sx 1 = 'Male'

2 = 'Female';

value et 1 = 'Non-Hispanic White'

2 = 'Non-Hispanic Black'

3 = 'Mexican American'

4 = 'Other';

value vet 1 = 'Non-African American'

2 = 'African American';

value vag 1 = 'Ages 20 to 70 years'

2 = 'Ages >= 70 years';

value yst 1 = '1999-2000'

2 = '2001-2002'

3 = '2003-2004'

4 = '2005-2006'

5 = '2007-2008'

6 = '2009-2010'

7 = '2011-2012';

value mn 0 = 'Regular (non-menthol)'

1 = 'Menthol';

value edu 1 = 'Less Than 9th Grade';

2 = '9-11th Grade (Includes 12th grade with no diploma)'

3 = 'High School Grad/GED or Equivalent'

4 = 'Some College or AA degree'

5 = 'College Graduate or above'

7 = 'Refused'

9 = 'Don't Know';

libname slib7 '\nhanes\2011-2012\Questionnaires\Smoking';

libname slib6 '\nhanes\2009-2010\Questionnaires\Smoking';

libname slib5 '\nhanes\2007-2008\Questionnaires\Smoking';

libname slib4 '\nhanes\2005-2006\Questionnaires\Smoking';

libname slib3 '\nhanes\2003-2004\Questionnaires\Smoking';

libname slib2 '\nhanes\2001-2002\Questionnaires\Smoking';

libname slib1 '\nhanes\1999-2000\Questionnaires\Smoking';

libname dlib7 '\nhanes\2011-2012\Demographics';

libname dlib6 '\nhanes\2009-2010\Demographics';

libname dlib5 '\nhanes\2007-2008\Demographics';

libname dlib4 '\nhanes\2005-2006\Demographics';

libname dlib3 '\nhanes\2003-2004\Demographics';

libname dlib2 '\nhanes\2001-2002\Demographics';

libname dlib1 '\nhanes\1999-2000\Demographics';

libname mlib7 '\nhanes\2011-2012\Questionnaires\MedicalConditions';

libname mlib6 '\nhanes\2009-2010\Questionnaires\MedicalConditions';

libname mlib5 '\nhanes\2007-2008\Questionnaires\MedicalConditions';

libname mlib4 '\nhanes\2005-2006\Questionnaires\MedicalConditions';

libname mlib3 '\nhanes\2003-2004\Questionnaires\MedicalConditions';

libname mlib2 '\nhanes\2001-2002\Questionnaires\MedicalConditions';

libname mlib1 '\nhanes\1999-2000\Questionnaires\MedicalConditions';

libname plib7 '\nhanes\2011-2012\Questionnaires\BloodPressure';

libname plib6 '\nhanes\2009-2010\Questionnaires\BloodPressure';

libname plib5 '\nhanes\2007-2008\Questionnaires\BloodPressure';

libname plib4 '\nhanes\2005-2006\Questionnaires\BloodPressure';

libname plib3 '\nhanes\2003-2004\Questionnaires\BloodPressure';

libname plib2 '\nhanes\2001-2002\Questionnaires\BloodPressure';

libname plib1 '\nhanes\1999-2000\Questionnaires\BloodPressure';

libname elib7 '\nhanes\2011-2012\Exams';

libname elib6 '\nhanes\2009-2010\Exams';

libname elib5 '\nhanes\2007-2008\Exams';

libname elib4 '\nhanes\2005-2006\Exams';

libname elib3 '\nhanes\2003-2004\Body Measurements';

libname elib2 '\nhanes\2001-2002\Exams';

libname elib1 '\nhanes\1999-2000\Exams';

options nocenter fmtsearch=(fin);

data demo; set dlib1.demo (in=in1)

dlib2.demo_b (in=in2)

dlib3.demo_c (in=in3)

dlib4.demo_d (in=in4)

dlib5.demo_e (in=in5)

dlib6.demo_f (in=in6)

dlib7.demo_g (in=in7);;

if in1 then yset = 1;

if in2 then yset = 2;

if in3 then yset = 3;

if in4 then yset = 4;

if in5 then yset = 5;

if in6 then yset = 6;

if in7 then do;

ridageex = ridexagm;

yset = 7;

end;

if ridreth1 in (1) then eth = 3;

if ridreth1 in (2,5) then eth = 4;

if ridreth1 = 3 then eth=1;

if ridreth1=4 then eth=2;

if yset in (5,6,7) then indhhinc = indhhin2;

keep seqn riagendr ridageyr eth sdmvstra sdmvpsu wtint2yr wtmec2yr yset

dmdeduc2 indhhinc indfmpir ridageex ridreth1 wtmec4yr wtint4yr;

proc sort; by yset seqn;

data mc; set mlib1.mcq (in=in1)

mlib2.mcq_b (in=in2)

mlib3.mcq_c (in=in3)

mlib4.mcq_d (in=in4)

mlib5.mcq_e (in=in5)

mlib6.mcq_f (in=in6)

mlib7.mcq_g (in=in7);

if in1 then yset = 1;

if in2 then yset = 2;

if in3 then yset = 3;

if in4 then yset = 4;

if in5 then yset = 5;

if in6 then yset = 6;

if in7 then yset = 7;

if mcq160F in (1,2) then stroke = mcq160F;

if mcq160E in (1,2) then MI = mcq160E;

if mcq160B in (1,2) then chf = mcq160B;

if mcq160G in (1,2) then COPD = mcq160g;

if COPD in (2,.) and MCQ160K in (1,2) then COPD = mcq160k;

keep yset seqn stroke MI CHF COPD;

proc sort; by yset seqn;

data bp; set plib1.bpq (in=in1)

plib2.bpq_b (in=in2)

plib3.bpq_c (in=in3)

plib4.bpq_d (in=in4)

plib5.bpq_e (in=in5)

plib6.bpq_f (in=in6)

plib7.bpq_g (in=in7);

if in1 then yset = 1;

if in2 then yset = 2;

if in3 then yset = 3;

if in4 then yset = 4;

if in5 then yset = 5;

if in6 then yset = 6;

if in7 then yset = 7;

if bpq020 in (1,2) then hypertension = bpq020;

keep yset seqn hypertension;

proc sort; by yset seqn;

data exam; set elib1.bmx (in=in1)

elib2.bmx_b (in=in2)

elib3.bmx_c (in=in3)

elib4.bmx_d (in=in4)

elib5.bmx_e (in=in5)

elib6.bmx_f (in=in6)

elib7.bmx_g (in=in7);

if in1 then yset = 1;

if in2 then yset = 2;

if in3 then yset = 3;

if in4 then yset = 4;

if in5 then yset = 5;

if in6 then yset = 6;

if in7 then yset = 7;

keep seqn bmxbmi yset;

proc sort; by yset seqn;

data smk1; set slib1.smq (in=in1)

slib2.smq_b (in=in2);

if in1 then yset = 1;

if in2 then yset = 2;

if smd090 > 0 and smd090 <= 95 then avgsmk = smd090;

if avgsmk = . and smd070 >=1 and smd070 < 700 then avgsmk = smd070;

if smd030 > 0 and smd030 <= 77 then smkage=smd030;

IF SMD080 >= 1 and SMD080 <= 30 then smkdays = SMD080;

if smq020 = 1 and smq040 in (1,2) then smoker = 1; else smoker = 2;

if smoker = 2 and smd080 >=1 and smd080 <= 30 then smoker = 1;

if smoker = 2 and smd070 >=1 and smd070 < 777 then smoker = 1;

if smd075 >= 1 and smd075 <= 71 then smkyrs = smd075;

other = 0;

if smq140 in (1,2) or smq170 in (1,2) or smq200 in (1,2) or smq230 in (1,2) then other = 1;

keep seqn smoker yset avgsmk smkdays smkage smd100mn smkyrs smq020 smq040 smd092 smdUPCA smd070 other;

rename smd100mn=menthol;

data smk2; set slib1.smqmec (in=in1)

slib2.smqmec_b (in=in2)

slib3.smqmec_c (in=in3);

if in1 then yset = 1;

if in2 then yset = 2;

if in3 then yset = 3;

if SMQ664b=1 OR SMQ664c=1 or smq664m=1 OR

smq664o = 1 or smq664w=1 OR

(SMQ666K IN (1,2,3) OR SMQ666S IN (1,2,3)) then menthol = 1;

if menthol = . and (SMQ664b=2 OR SMQ664c=2 or smq664m=2 OR

smq664o = 2 or smq664w=2 OR

(SMQ666b IN (1,2,3) OR SMQ666c IN (1,2,3) or

SMQ666m IN (1,2,3) OR smq666N in (1,2,3) OR

SMQ666o IN (1,2,3) OR SMQ666w IN (1,2,3))) then menthol = 0;

IF SMQ640 >= 1 and smq640 <= 30 then smoker=1; else smoker = 2;

if SMQ650 >= 1 and SMQ650 < 77 then avgsmk = SMQ650;

if avgsmk = . and smd720 > 0 and smd720 < 777 then avgsmk = smd720;

IF SMQ640 >= 1 and SMQ640 <= 30 then smkdays = SMQ640;

if smkdays = . and smd710 > 0 and smd710 <=5 then smkdays = smd710*6;

if smd630 >=6 and smd630<=19 then smkage=smd630;

keep seqn smoker yset avgsmk smkdays smkage menthol smq640;

data smk3; set slib3.smq_c (in=in3)

slib4.smq_d (in=in4)

slib5.smq_e (in=in5)

slib6.smq_f (in=in6)

slib7.smq_g (in=in7);

if in3 then yset = 3;

if in4 then yset = 4;

if in5 then yset = 5;

if in6 then yset = 6;

if in7 then yset = 7;

rename smd100mn=menthol;

keep seqn smd030 smq050Q smq050U smq660

smd055 smd650 smd100mn smd630 smq670 smq664m smq664C SMQ664w SMQ664b

smq664o smq666K SMQ666S SMd641 smq666b smq666c smq666m smq077 smd070 smq020

smq666o smq666w smq670 yset smq040 smq050Q smq050U smd075 smd093 SMDUPCA SMD070;

data smk3; set smk3;

if menthol = . then do;

if SMQ664b=1 OR SMQ664c=1 or smq664m=1 OR smq660 in (4,7) or

smq664o = 1 or smq664w=1 OR

(SMQ666K IN (1,2,3) OR SMQ666S IN (1,2,3)) then menthol = 1;

if menthol = . and (SMQ664b=2 OR SMQ664c=2 or smq664m=2 OR

smq664o = 2 or smq664w=2 OR smq660=3 or

(SMQ666b IN (1,2,3) OR SMQ666c IN (1,2,3) or

SMQ666m IN (1,2,3) OR

SMQ666o IN (1,2,3) OR SMQ666w IN (1,2,3))) then menthol = 0;

end;

IF SMd641 >= 1 and smd641 <= 30 then smkdays = SMD641;

if smd630 >=6 and smd630<=19 then smkage=smd630;

if smd030 >0 and smd030<=85 then smkage=smd030;

if smd075 >= 1 and smd075 <= 71 then smkyrs = smd075;

if smd650 >0 and smd650 < 777 then avgsmk = smd650;

if avgsmk = . and smd070 >= 1 and SMD070 < 777 then avgsmk = SMD070;

if smq020 = 1 and smq040 in (1,2) then smoker = 1; else smoker = 2;

if smq020 = . and smq040 = . and (smd641 >=1 and smd641<=30) then smoker = 1;

if smd075 >= 1 and smd075 <= 71 then smkyrs = smd075;

if smoker = 2 and smd641 >=1 and smd641 <= 30 then smoker = 1;

if smoker = 2 and smd070 >=1 and smd070 < 777 then smoker = 1;

keep seqn smoker yset avgsmk smkage menthol smkdays smkyrs smd093 smdUPCA smd070 smd641 smq040;

data smk4; set slib3.smq_c;

yset = 3;

other = 0;

if smq140 in (1,2) or smq170 in (1,2) or smq200 in (1,2) or smq230 in (1,2) then other = 1;

Keep yset other seqn;

data smk5; set slib4.smqrtu_d (in=in4)

slib5.smqrtu_e (in=in5)

slib6.smqrtu_f (in=in6)

slib7.smqrtu_g (in=in7);

if in4 then yset = 4;

if in5 then yset = 5;

if in6 then yset = 6;

if in7 then yset = 7;

other = 0;

if smq690B = 2 or smq690C = 3 or smq690D = 4 or smq690E = 5 then other = 1;

Keep yset other seqn;

data smoking; set smk1 smk2 smk3;

proc sort; by yset seqn;

data sother; set smk4 smk5;

proc sort; by yset seqn;

data fin.all; merge smoking demo exam mc bp sother; by yset seqn ;

format menthol mn. eth et. riagendr sx. ceth vet. vage vag. other yn.;

if smoker < 0 then smoker = 2;

if smkage >= 0 and ridageyr >= 0 and ridageyr >= smkage then pkyrs = avgsmk/20.0 * (ridageyr - smkage+1);

else do;

if smkyrs > 0 then pkyrs = avgsmk/20.0 * smkyrs;

else do;

pkyrs = avgsmk/20.0;

end;

end;

if ridageyr >= 70 then vage = 2;

if ridageyr >= 20 and ridageyr < 70 then vage = 1;

if eth = 2 then veth = 2;

if eth in (1,3,4) then veth = 1;

wgt = wtint2yr;

label Smkage = 'age started smoking (smd030 for 20+ smd630 for <20)'

avgsmk = 'avg cig smoked per day in last 30 days'

other = 'Uses other tobacco products (0 = no, 1 = yes)';

/*Example of SAS code for Hypertension using NHANES 1999-2012 data and the Rostron covariates. */

DATA tst; SET fin.all;

format menthol mn.;

if yset in (1,2,3,4,5,6,7);

wgt = wtint2yr/7;

output tst;

data ros; set tst;

if yset in (1,2,3,4,5,6,7) and smoker=1 and ridageyr >=20 and menthol in (0,1)

and pkyrs >= 0 and bmxbmi >= 0 and indfmpir >= 0;

proc freq data=ros;

ods output crosstabfreqs=atab;

table menthol*hypertension /norow nocol nopercent nocum;

table riagendr*menthol*hypertension /norow nocol nopercent nocum;

table eth*menthol*hypertension /norow nocol nopercent nocum;

table vage*menthol*hypertension /norow nocol nopercent nocum;

proc Surveylogistic data=ros;

ods output oddsratios=odds1;

ods output ResponseProfile = num;

strata sdmvstra;

cluster sdmvpsu;

weight wgt;

class hypertension eth riagendr

menthol(param=ref ref=last) ;

model hypertension = menthol riagendr ridageyr eth pkyrs bmxbmi indfmpir ;
